# Palmoplantar keratoderma is associated with esophagus squamous cell cancer in Van region of Turkey: a case control study

**DOI:** 10.1186/1471-2407-5-90

**Published:** 2005-07-28

**Authors:** Mahmut Ilhan, Tugrul Erbaydar, Necmettin Akdeniz, Sevket Arslan

**Affiliations:** 1 Medical Oncology Unit of Internal Medicine Department, School of Medicine, Yuzuncu Yil University, Van, Turkey; 2 Public Health Department, School of Medicine, Yuzuncu Yil University, Van, Turkey; 3 Dermatalogy Department, School of Medicine, Yuzuncu Yil University, Van, Turkey; 4 Internal Medicine Department, School of Medicine, Yuzuncu Yil University, Van, Turkey

## Abstract

**Background:**

Esophagus squamous cell cancer (ESCC) is the most common cancer in women with 20.2% and second in men with 10.7% relative frequency among all cancer cases diagnosed in Van Region in the east of Turkey. Ninety percent of all esophageal cancer cases are ESCC and 20–30% of them have family history of esophageal cancer. The most clear defined hereditary predisposition associated with ESCC is palmoplantar keratoderma (PPK). To examine the relationship between ESCC and PPK, we have carried out this case control study.

**Methods:**

The case group consisted of 48 subjects who had new diagnosis of ESCC and did not receive any chemo or radiotherapy. The control group consisted of 96 healthy individuals who were visitors of their relatives in the hospital. Two control persons who matched for age, gender, living place (urban /rural) and region were selected for each case. All subjects were evaluated for PPK by dermatologist. Evaluation was graded as none, mild, evident and severe. None and mild subjects were classified as negative for PPK; and others as positive. Relationship between ESCC and PPK was evaluated with odds ratios and confidence intervals for cases with or without family history of ESCC.

**Results:**

The PPK frequencies were 92.3% in ESCC cases with family history, 62.5% in ESCC cases without family history, 70.8% in all ESCC cases, and 28.1% in the control group. Odds ratios for cases with or without family history of esophageal cancer, and for the whole case group were found as 30.7 (95%CI = 3.8–247.4), 4.3 (95%CI = 1.9–9.8) and 6.2 (95%CI = 2.9–13.3) respectively.

**Conclusion:**

Presence of PPK lesions represents genetic susceptibility for ESCC. This susceptibility for ESCC is the highest among those who have PPK lesions and a positive family history of esophageal cancer. Furthermore, a PPK sufferer has an increased risk of developing ESCC even if there is no family history of esophageal cancer.

## Background

Esophageal cancer is the eighth most common cancer and the sixth most common cause of cancer-related mortality in the world [1]. Esophagus cancer is not a common type of cancer in Turkey but it remains as a frequently diagnosed type in Van region in the east of Turkey [2,3]. Esophagus cancer is the most common cancer among women with a proportion of 20.2% and the second most common cancer among men with a proportion of 10.7% among all diagnosed cancer cases in Van region [3]. Histologically, approximately 90% of esophagus cancer cases in the region are squamous cell carcinoma.

**Figure 1:**
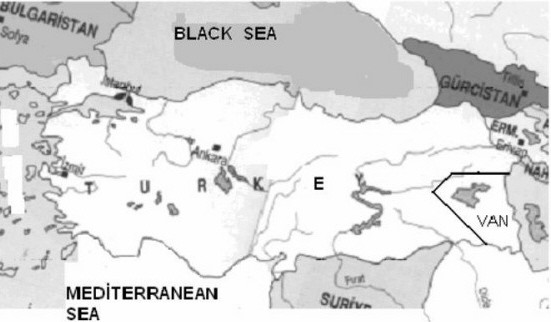
The Van region is located around Van Lake in the east of Turkey.

The Van region, which is located around the Van lake in the east of Turkey has a population around 2 million people which were settled mostly in rural areas (Figure 1).

A series of case control studies from the regions where ESCC is frequent indicate that smoking, alcohol consumption, hot foods and beverages, and insufficient intake of fruits and vegetables are significant risk factors for ESCC [4,5]. The same factors except alcohol consumption has been identified as risk factors in two previous studies in Van region [6,7]. In addition to these environmental-acquired factors, hereditary – familial risk factors for ESCC has been described. The most clear hereditary predisposition syndrome associated with ESCC is tylosis. Tylosis is a hereditary autosomal dominant syndrome. The most common sign of this syndrome is palmoplantar keratoderma (PPK), which develops in pressure-exposed areas of cutis [8]. PPK is an abnormal thickening and callusing of the palms and soles due to abnormal keratinisation [9]. Additionaly, oral leukoplakia and esophageal hyperkeratosis may accompany to palmoplantar keratoderma. Association between tylosis and ESCC was clearly proven in three good-defined pedigrees [10-12]. In these families, the risk of developing ESCC among individuals who had PPK was reported as 40–90% until the age of 70 years; and the risk of developing ESCC was very high compared to the family members who had no PPK within the same family. Although familial aggregation in ESCC was reported in a variety of studies in the literature [13-15], it is not known whether the frequency of PPK in ESCC cases is high in comparison to healthy individuals. According to our observations, 20–30% of ESCC patients have a family history of esophageal cancer in their relatives.

We designed this case control study to examine the hereditary predisposition in ESCC, by determining the PPK frequency among ESCC cases with or without family history of esophageal cancer in comparison with the control group including healthy individuals.

## Methods

This study was conducted between December 2003 and December 2004 in the Yuzuncu Yil University Medical School Hospital. We designed the case control study to investigate the proportions of PPK lesions among ESCC patients in comparison to healthy individuals.

The case group consisted of 48 subjects who had new diagnosis of ESCC. ESCC diagnosis was done by endoscopic biopsy. Patients who received any previous chemo or radiotherapy program were not included in the case group because of probability that chemo and radiotherapy could interfere cell dividing kinetics of the epidermis and could suppress keratoderma. Three ESCC cases were also excluded because they had striking and intensive tinea pedis associated to PPK. Two subgroups of the case group were identified according to their family history of esophageal carcinoma. The cases who have at least one additional esophagus cancer case among their first degree relatives were accepted as family history positive ESCC cases, and the rest of the group was accepted as family history negative ESCC cases.

The control group consisted of 96 healthy individuals who were selected among the visitors of patients hospitalized at the departments of internal medicine in the Yuzuncu Yil University Medical School Hospital. At first, the visitors who were randomly selected at the inpatient clinics were interviewed by an introductory questionnaire. Then, those who have no diagnosed cancer and no close relative having esophageal or gastric cancer were accepted to be eligible for the control group.

The control group was matched for age group, gender, living place (urban /rural) and region of birth (Van region or else). For each person in the case group, two persons who match for age, gender, living place and region of birth were selected among the eligible persons for the control group. We intended to decrease the random error by selecting two controls for each case, instead of one by one matching. Consequently, the control group was precisely matched with the case group for the criteria that might influence the probability of having PPK.

Clinical examination of the cases and the controls were done by consulting a dermatologist to identify PPK lesions. Oral leukoplakia, which is a rare finding of tylosis, was not investigated. Palmoplantar lesions were graded as no PPK, mild PPK, evident PPK and severe PPK. Those who have no or mild PPK lesions were classified as PPK negative, and those who have evident or severe PPK lesions were classified as PPK positive. According to the type of PPK and the involved area, PPK positive subjects were further classified as diffuse or focal and palmar, plantar or palmoplantar PPK.

Statistical analysis was performed with the SPSS (11.5) software. Odds ratio, 95% confidence interval (CI) of the odds ratio, and chi-square statistics were calculated for the whole case group, and the subgroups in comparison to the controls. The type and localization of PPK lesions in the case and the control groups were compared with chi-square statistics.

## Results

The distribution of the cases and the controls according to the matching criteria and the clinical characteristics of the ESCC cases are shown in table 1.

Table 2 presents the comparison of the case and control groups according to the proportions of PPK positive subjects. In the case group, the frequency of PPK positive subjects was 70.8% while it was 28.1% in the control group. The odds ratio of accompanying PPK to ESCC was calculated as 6.2 (95%CI = 2.9 – 13.3), and the difference between the proportions of PPK in the case and control groups was highly significant (p < 0.001).

Table 2 also presents the comparisons between the subgroups of cases and the controls. The prevalence of PPK were 92.3% in ESCC cases with family history, and 62.5% in ESCC cases without family history. In all comparisons, the association between PPK and ESCC was statistically significant. The odds ratio of having PPK for the cases with family history was found noticeably high (OR = 30.7; 95%CI = 3.8–247.4).

Table 3 presents the type and localization of PPK lesions in the case and control groups. In both groups, the type of PPK lesions was mostly diffuse and the localization was plantar. Although the proportion of focal and palmar lesions was higher in the control group compared to the cases; the difference between two groups was not statistically significant by chi-square statistics (p > 0.05).

## Discussion

Our results showed a significant relationship between PPK and ESCC. The relationship is particularly high for ESCC cases with family history of esophageal cancer. As presented in Table 2, the proportion of PPK is higher in the ESCC group (70.7%) compared with the control group (28.1%) with OR = 6.2 (95%CI = 2.9–13.3). The proportion of PPK is strikingly high in cases with positive family history (92.3%).

The association of PPK with ESCC was firstly reported by Clarke and McConnel in one family in 1954 [16]. Then, this association was confirmed by Howel-Evans et al [10]. In their study of two related Liverpool families, esophageal carcinoma occurred in 18 of 48 tylotic family members, but only in one of 87 non-tylotic members. Later, Ellis et al. reviewed these two related families and portrayed a single family containing 345 individuals, 92 were diagnosed as having focal PPK of which 32 died, 21 of esophageal cancer [17]. In this pedigree, there was no oesophageal cancer in family members who were unaffected by PPK. It was calculated that a PPK sufferer in this pedigree had a 92% probability of dying from oesophageal cancer by age of 70 [17]. Subsequently, a large midwestern American pedigree containing 125 affected individuals with an identical pattern of PPK was described, in which the skin disorder was associated with oesophageal and other cancer [11]. In American pedigree, there were 19 reported malignancies in members with PPK, eight of which were esophageal or oral squamous cell carcinomas. The life time risk of developing oesophageal cancer for an affected member of this family was calculated to be 40% by age 70. Maillefer and Greydanus reported that late-onset PPK lesions (Type A) have a significant association with ESCC, while early onset PPK lesions (Type B) have no such association [18].

We suggest that, the underlying reason of the significant association between ESCC and PPK in our study is a hereditary predisposition which causes both PPK lesions and ESCC. While there is evidence that late onset PPK might be a paraneoplastic finding for some other types of cancer [19-21], our observations do not confirm such explanation for the association between PPK and ESCC. In these studies [19-21], PPK lesions were found to be paraneoplastic findings because these lesions were regressed and disappeared if the cancer was treated and response was achieved. In our study, we observed some cases that, there was no change regarding the PPK lesions after esophageal cancer symptoms regressed and disappeared following surgery and primary curative chemoradiotherapy. Although we do not have reliable data on the onset of PPK lesions in most ESCC patients, a few of them exactly remembered that PPK lesions occurred 15–20 years before ESCC symptoms. Maillefer and Greydanus reported that the onset of type A tylosis occur between the ages of 5–15 years, while the onset of esophageal cancer occur no less than the age of 29 years. Therefore, we suggest that PPK lesions do not develop as a result of ESCC development; rather, ESCC and PPK develop on the same hereditary background.

PPK may also be caused by environmental reasons and lifestyle [22]. Although we did not matched the two groups for the occupation, we controlled the effects of age, gender and living place (urban / rural). Eighty-eight percent of both groups consisted of men living in rural areas and women in rural and urban areas. Another survey conducted in Van region showed that almost all women (98%) in Van were housewives [23]; thus, we may suggest that women in the case and control groups have similar lifestyles. Almost all men who live in rural areas work in farming and breeding; so, they have also very similar lifestyles both in the case and control groups. Thus, we assume that there is no considerable lifestyle difference between cases and controls.

The proportion of PPK among the ESCC cases without family history (62.5%) was also significantly higher than that of controls (28.1%); and the odds ratio was 4.3 (95%CI = 1.9–9.8). In other words, presence of PPK can make up risk for ESCC in future even if family history of ESCC is not present. To explain this result, we need to look at the genetic background of PPK and ESCC. The suspected gene for PPK and ESCC couldn't be isolated exactly until now. However, the locus for focal PPK in association with ESCC has been mapped on chromosome 17q25. This gene region frequently undergoes to deletion in hereditary as well as in sporadic ESCC cases [24,25]. The loss of heterozygosity on 17q25 was detected in 71% and 69% of sporadic ESCC cases. Therefore, it was proven that this chromosomal region is also commonly affected in sporadic ESCC cases. The reason why we observed PPK also in ESCC cases without family history may depend on the affected same gene locus.

Even though the lowest proportion of PPK in our study was in the control group, it is still a considerably high proportion (28.1%) compared to some other studies. In their review on PPK, Maillefer and Greydanus described PPK as a rare syndrome [18]. The prevalence of PPK in a region of Sweden, where PPK is frequently observed, has been reported to be 0.3–0.55% by two studies [26,27]. We may consider the high proportion of PPK in our control group as a sign of high PPK prevalence in Van region. One reason of this high prevalence may be work styles that involve a lot of standing/walking which is highly prevalent in both the rural and urban areas in Van region. Another reason may be the genetic characteristics of the people. We noted above that all of our ESCC cases and controls were people whose places of birth were Van region. Due to a series of social and economical reasons, people living in this region frequently migrate to other regions of Turkey; but there is almost no migration from other regions of Turkey to Van region. Traditionally, most of the marriages in the local communities happen between relatives, and a part of these marriages happen between cousins. Consequently, the region has a homogeneous genetic population to a degree and therefore, hereditary syndromes can be frequently observed in the local community as in hereditary PPK cases. These characteristics, perhaps, made Van region a suitable place to observe such association between ESCC and PPK.

The study contains some limitations that should be noted. Firstly, sizes of the case and control groups are not large. This could lead to a type II error if there was not a big difference between the proportions in the case and control groups. In our study, the small group sizes did not lead to insignificant results, but this caused some large confidence intervals as seen in table 2. Secondly, our data on the association between PPK and ESCC is based on a matched case control design; rather than longitudinal. This limits the extent to which conclusions about causality can be drawn. Thirdly, we did not investigate the genetic inheritance of the PPK in relatives of ESCC patients and controls; thus, we are not able to discuss the existence of a dominant inheritance pattern for PPK. This point should be a focus of future research.

## Conclusion

We suggest that presence of PPK lesions represents genetic susceptibility for ESCC. This susceptibility for ESCC is highest among those who have PPK lesions and a positive family history of esophageal cancer. Furthermore, a PPK sufferer has an increased risk of developing ESCC even if there is no family history of esophageal cancer. This result provides additional evidence on the genetic background of PPK and ESCC.

## Competing interests

The author(s) declare that they have no competing interests.

## Authors' contributions

MI conceived of the study, and participated in its design, the statistical analysis, and coordination and drafted the manuscript

TE participated in the design of the study and performed the statistical analysis and helped to draft the manuscript

NA helped for dermatologic evaluation of subjects and participated in design of the study

SA helped for acquisition of data and participated in design of the study

All authors read and approved the final manuscript.

## Pre-publication history

The pre-publication history for this paper can be accessed here:



## Figures and Tables

**Table 1:** The general characteristics of cases and controls

Characteristics	Case group(n = 48)	Control group(n = 96)
Age		
Mean (± sd)	50.7 (± 12.0)	49.8 (± 11.5)
Range	25 – 72	24 – 75
Sex		
Female	30 (62.5%)	60 (62.5%)
Male	18 (37.5%)	36 (37.5%)
The living place		
Rural	27 (56.3%)	54 (56.3%)
Urban	21 (43.8%)	42 (43.8%)
The region of birth		
Van region	48 (100%)	96 (100%)
Other	0	0
Stage in time of admission		
Localized	17 (35.4%)	
Locally-advanced	23 (47.9%)	
Metastatic	8 (16.7%)	
Histological type		
SCC	48 (100%)	
Adenocarcinoma	0	

SCC: Squamous cell carcinoma

**Table 2:** Comparison of the case and the control groups according to PPK^a ^lesions

Group	PPK Positive			
			
	n	%	OR (95%CI)	p
All Cases (n = 48)^b^	34	70.8	6.2 (2.9–13.3)	<0.0001
Controls (n = 96)	27	28.1		
				
Cases with FH^c^(n = 13)	12	92.3	30.7 (3.8–247.4)	<0.0001
Controls (n = 96)	27	28.1		
				
Cases without FH^d ^(n = 32)	20	62.5	4.3 (1.9–9.8)	<0.0001
Controls (n = 96)	27	28.1		
				
Cases with FH (n = 13)	12	92.3	7.2 (0.83–62.6)	0.046
Cases without FH (n = 32)	20	62.5		

a : Palmoplantar keratoderma

b: Because family history of three cases were missing, sum of cases with and without FH is not equal to all cases

c: Cases who have at least one esophagus cancer case among their first degree relatives

d: Cases who have no history of esophagus cancer among their first degree relatives

**Table 3:** The type and localization of PPK^a ^lesions in the case and the control groups

Characteristics of PPK	Cases with PPK (n = 34)		Controls with PPK (n = 27)	
	
	n	%	n	%
Pattern of keratoderma				
Diffuse	32	94.1	21	77.8
Focal	2	5.9	6	22.2
Involved area				
Palmar	0	0	2	7.4
Plantar	27	79.4	15	55.6
Palmoplantar	7	20.6	10	37.0

a : Palmoplantar keratoderma
